# Application of Adaptive Wave Cancellation Underwater to a Piezoelectric-Material-Based Multilayer Sensor

**DOI:** 10.3390/s20010134

**Published:** 2019-12-24

**Authors:** Hyodong Lee, Hwijin Park, Kwan Kyu Park, Hak Yi

**Affiliations:** 1 Department of Mechanical Engineering, Kyungpook National University, Daegu 41566, Korea; 2 Department of Robot Engineering, Kyungpook National University, Daegu 41566, Korea; 3 Department of Mechanical Engineering, Hanyang University, Seoul 133791, Korea; kwankyu@hanyang.ac.kr

**Keywords:** piezoelectricity, multilayer sensor, active noise canceling, underwater, adaptive gain control

## Abstract

This paper concerns the use of adaptive wave cancellation in a new multilayer smart skin sensor to attenuate the primary low-frequency noise underwater. The proposed multilayered system is designed with a piezoelectric actuator (Pb(In_1/2_Nb_1/2_)O_3_–Pb(Mg_1/3_Nb_2/3_)O_3_–PbTiO_3_ composite) and two layers of polyvinylidene fluoride to accelerate wave absorption. Furthermore, the use of a combination of an adaptive control scheme and a time-delay signal separation method has the potential to provide the proposed absorber system with a wave cancellation capability and thereby enable the absorber system to respond to environmental changes underwater. The use of smart piezoelectric materials and an adaptive control approach enables the absorber system to achieve the high attenuation level of the reflected waves, unlike typical absorber systems based on active noise control. Echo reduction experiments showed that the proposed piezoelectric-based multilayer sensor with an adaptive controller could attenuate reflected wave signals effectively.

## 1. Introduction

Since the 1950s, underwater acoustic absorbers have typically been passive systems employing energy-absorbing materials to block the transmission of acoustic waves. These passive absorbers satisfactorily attenuate undesirable high-frequency signals. However, they have a drawback, becoming bulky when designed to cancel relatively low-frequency signals. Since the use of passive materials for the cancellation of low-frequency signals has been very limited, active noise control has attracted considerable attention [1,2].

For the achievement of active noise control and the creation of a silent space, a piezoelectric material is used to measure the incident waves and generate control waves with the opposite phase for canceling the incident waves. In particular, active noise control has the following advantages: The physical size of the absorber system is less limited to the frequency and acoustic absorption in the broadband frequency range by a digital controller is possible [1,2,3,4,5,6].

To enhance the performance of the active noise control system, many investigations have been conducted by considering a variety of piezoelectric materials [7,8,9]. However, the use of the composite material Pb(In_1/2_Nb_1/2_)O_3_–Pb(Mg_1/3_Nb_2/3_)O_3_–PbTiO_3_ (PIN–PMN–PT) for active noise cancellation has not been examined yet. This composite material is a single-crystal material with high transmitting/receiving sensitivity. Furthermore, the PIN–PMN–PT composite shows a good transmitting/receiving performance, with a larger broadband frequency than any other piezoelectric materials. An advantage of the composite material is that it is capable of wave cancellation over a large frequency range underwater [10,11,12]. In an acoustic absorber system with a single sensor, a common transmission path for both incident and reflected waves renders the separate analysis of the two types of waves difficult. To overcome this difficulty and to facilitate the use of a time-delay signal separation method, the present study considered an acoustic absorption system with two receiving sensors [13,14].

Recently, researchers have focused on an acoustic absorber, called smart skin, installed on the surface of a variety of underwater control system [15,16]. The acoustic absorber mimics a smart sensor attached to the surface of human skin. This study proposes a smart skin comprising piezoelectric materials, two receiving sensors, and an intelligent digital controller. The smart skin shows higher echo reduction performance compared with other acoustic absorber systems. The multi-sensor system can separate superposed signals by using a time-delay signal separation method [10]. Furthermore, this study presents an application of an adaptive control scheme that can enable the multi-sensor system to achieve perfect noise cancelation when environmental changes occur.

This paper is organized as follows. Section 2 describes the proposed multilayer absorber system, and Section 3 discusses the transmitting and receiving sensitivities of the acoustic absorber system. Section 4 presents a noise cancellation strategy for the multilayer acoustic absorber system. Section 5 explains the proposed adaptive wave cancellation technique employed by the developed sensor system in underwater applications. Section 6 details the results of echo reduction experiments conducted in this study. Finally, Section 7 summarizes the conclusions.

## 2. Components of the Multilayer Acoustic Absorber System

### 2.1. System Design

**Figure 1:**
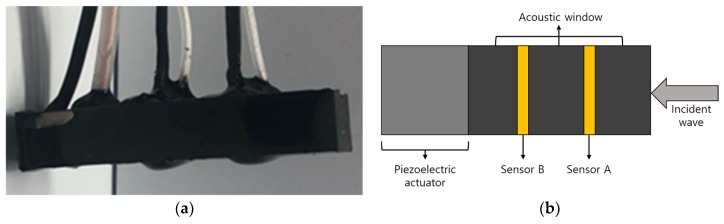
Smart skin acoustic absorber system: (**a**) the fabricated sensor and (**b**) the concept of the sensor.

As shown in Figure 1, the proposed multilayer acoustic absorber system comprised a piezoelectric-material-based actuator, multiple acoustic sensors, and multiple acoustic windows. Figure 1a,b show a fabricated multilayer acoustic absorber and its conceptual diagram, respectively. The dimensions of the sensor were 8 mm × 8 mm × 49.12 mm. In this study, the PIN–PMN–PT composite, which has high transmitting sensitivity, was used as a piezoelectric actuator. Its role was to provide a control wave with the opposite phase for canceling waves reflected from the surface of the piezoelectric material in the sensors. Rho-c rubber, whose acoustic impedance is similar to that of water, was used as the acoustic window. Finally, two layers of polyvinylidene fluoride (PVDF) were used to measure the superposition of the incident wave, reflected wave, and control wave, respectively. The receiving sensors (PVDF) were placed in front of the actuator to measure acoustic signals superposed on the incident and reflected signals.

Table 1 presents the properties of the materials used in the proposed multilayer acoustic absorber [17,18].

### 2.2. Fabrication

**Figure 2:**
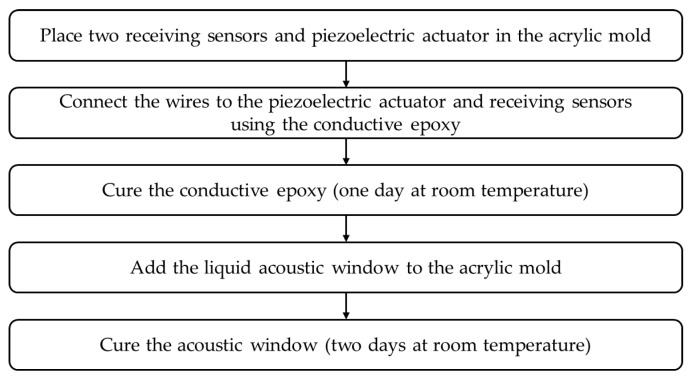
Fabrication process of the multilayer acoustic absorber.

**Figure 3:**
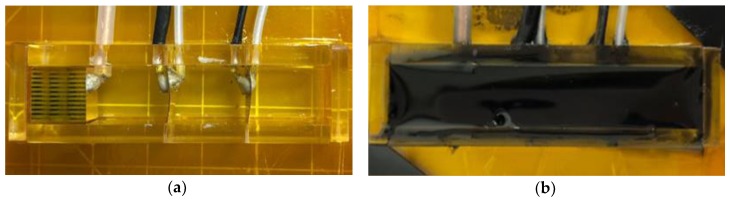
Fabrication of the proposed absorber: (**a**) the acrylic mold and (**b**) the liquid acoustic window.

Figure 2 shows the fabrication process of the multilayer acoustic absorber. The multilayer acoustic absorber, comprising two receiving sensors (PVDF), a piezoelectric actuator (PIN–PMN–PT composite), and an acoustic window (rho-c rubber), was fabricated using an acrylic mold. Its dimensions were about 14 mm × 11 mm × 55 mm. The piezoelectric actuator and receiving sensors were fixed in place on the acrylic mold, then the wires were connected to the piezoelectric actuator and receiving sensors using the conductive epoxy, as shown in Figure 3a. After curing the conductive epoxy, the liquid acoustic window was added to the mold and cured in the final step, as shown in Figure 3b.

## 3. Transmitting and Receiving Sensitivities of the Multilayer Acoustic Absorber System

It is necessary to determine the frequency dependence of the transmitting and receiving sensitivities of the proposed acoustic absorber system to achieve high acoustic absorption performance. The transmitting and receiving sensitivities obtained from a mathematical-model-based simulation and from experiments were compared.

The driving control signal of the piezoelectric actuator was given with a three-cycle sinusoidal signal. The installed hydrophone acted as a receiving sensor and collected the acoustic signal generated by the piezoelectric actuator. The estimated transmitting sensitivity T(ω) of the piezoelectric actuator in the multilayer sensor can be defined as

(1) $$T\left(\omega \right)=\frac{V_{output}}{V_{Input}\times S_{Hy}\left(\omega \right)},$$

where $S_{Hy}\left(\omega \right)$ is the receiving sensitivity of the hydrophone, and $V_{Output}$ and $V_{Input}$ are the hydrophone-measured signal and the drive control signal of the piezoelectric actuator, respectively.

With the measurement of the receiving sensitivity, the receiving sensitivity S(ω) was estimated from the measured amplitude of the acoustic signal received by the PVDFs:

(2) $$S\left(\omega \right)=\frac{V_{output}\times d}{V_{Input}\times T_{Pro}\left(\omega \right)},$$

where $T_{Pro}\left(\omega \right)$ is the transmitting sensitivity of the projector, d is the distance between the sensor and the projector, and $V_{Output}$ and $V_{Input}$ are the voltage signal received by the PVDF and the source signal of the projector, respectively.

**Figure 4:**
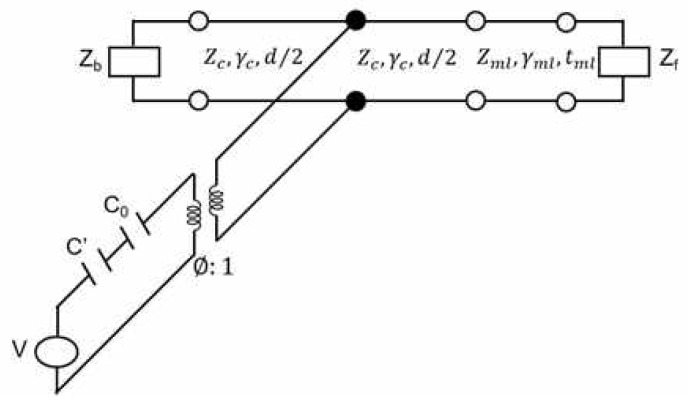
Equivalent circuit model of the proposed sensor.

In the simulation, the equivalent circuit model shown in Figure 4 was used [15,16]. The equivalent circuit model can be used to analyze a multilayer model and require less time compared to the finite element method. The calculation of the sensitivity depended on the properties of each material and the designed equivalent circuit model. In this study, the properties of each material, such as the Q factor (quality factor), thickness, and acoustic impedance, were based on the properties obtained from the experimental values. The transmitting/receiving sensitivity in the simulation was calculated using the equivalent circuit model equation [19,20,21,22].

**Figure 5:**
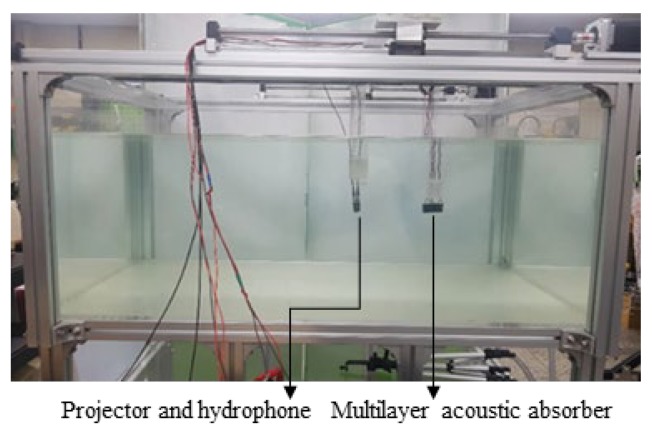
Water tank used in the experiment.

Experiments for measuring both sensitivities were conducted in a water tank that was 1 m long, 0.75 m wide, and 0.5 m high (Figure 5). The projector and hydrophone were positioned 150 mm from the front of the multilayer absorber system. A microcontroller (STM32H743ZI) and a digital-to-analog converter (AD5685R) were used to collect the transmitting and receiving sensitivities of the proposed acoustic absorber system.

**Figure 6:**
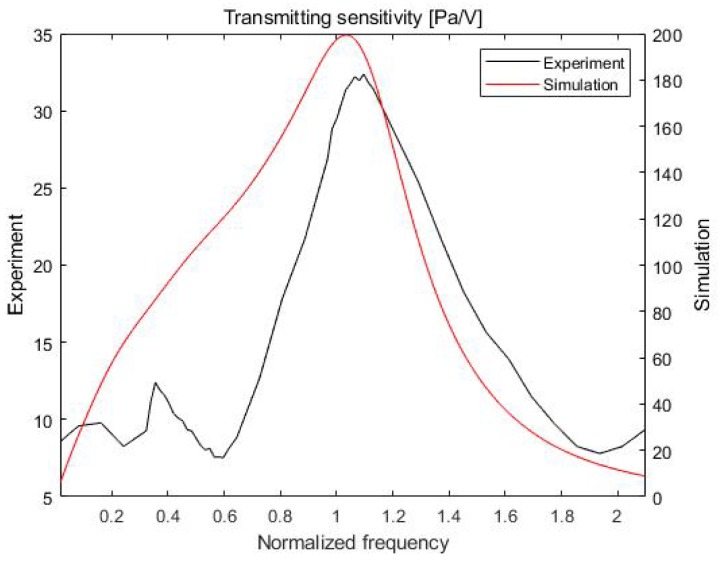
Transmitting sensitivity of the proposed actuator.

In Figure 6, the transmitting sensitivities obtained from the simulation and experiment show similar patterns. In particular, the transmitting sensitivity tends to decrease rapidly in regions far from the resonant frequency.

**Figure 7:**
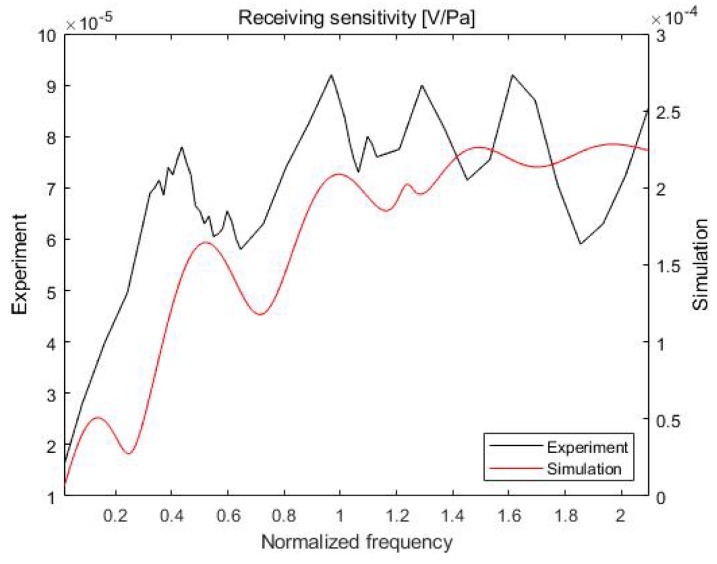
Receiving sensitivity of the proposed sensor.

Figure 7 shows the receiving sensitivities determined from the simulation and experiment plotted against the frequency. The variation of the receiving sensitivity with the frequency is similar in both the simulation and the experiment.

## 4. Design of the Active Control System for the Multilayer Acoustic Absorber System

The proposed control system has a function for separating a superposed signal into incident and reflected signals with a time-delay signal separation method, and it provides a driving control signal to the piezoelectric actuator for wave reduction.

### 4.1. Active Noise Control

**Figure 8:**
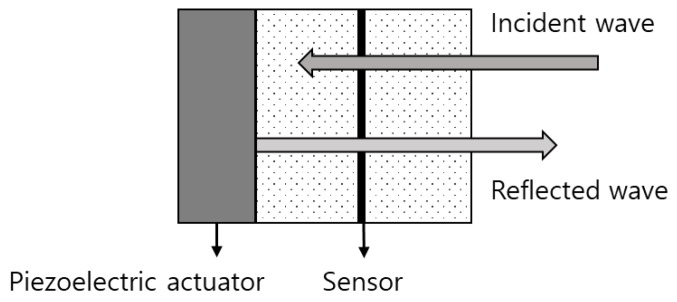
Concept of active noise control.

**Figure 9:**
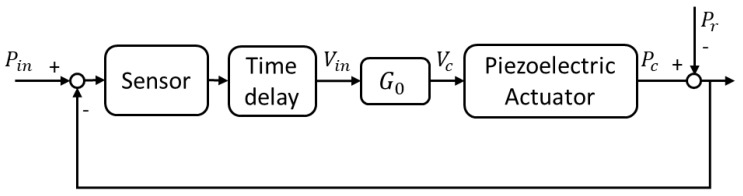
Block diagram of an active noise control scheme.

Active noise control mainly involves a piezoelectric system that attenuates the primary noise on the basis of the principle of superposition. Specifically, an anti-wave with the same amplitude as the primary noise signal, but with the opposite phase, is generated for canceling the primary noise, as shown in Figure 8. Figure 9 depicts a block diagram of a typical active noise control scheme.

(3) $$P_{r}+P_{c}=0.$$

The superposition of the reflected acoustic signal ($P_{r}$) and control acoustic signal ($P_{c}$) had to have zero amplitude, as shown in Equation (3), to achieve the active noise control performance required in this study.

### 4.2. Time-Delay Signal Separation Method

**Figure 10:**
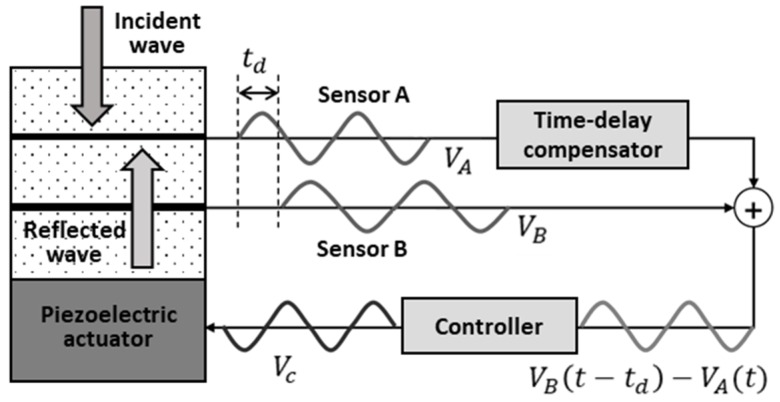
Concept of the multilayer acoustic absorber system.

Figure 10 shows a conceptual diagram of the control scheme of the smart skin acoustic absorber system. In this study, the incident wave was assumed to be a plane wave propagating perpendicular to the absorber system, with the reflected wave propagating in the opposite direction. The receiving sensors were assumed to be sufficiently thin and, therefore, the transmission loss could be neglected. The output voltages of sensors A $\left(V_{A}\right)$ and B ($V_{B}$) were estimated from the signal superposed on both the incident acoustic signal ($P_{in}$) and the reflected acoustic signal ($P_{r}$):

(4) $$V_{A}=S\left(\omega \right)\left[P_{in}\exp\left(jkr_{SS}+jkr_{SA}\right)+P_{r}\exp\left(-jkr_{SS}-jkr_{SA}\right)\right],$$

(5) $$V_{B}=S\left(\omega \right)\left[P_{in}\exp\left(jkr_{SA}\right)+P_{r}\exp\left(-jkr_{SA}\right)\right],$$

where S(ω) (in units of volt per pascal) is the receiving sensitivity of the sensors. The exponential terms represent the phases of the signals, and they depend on the relative positions of sensors A and B. k is the wave number of the acoustic signal. $r_{SS}$ is the distance between sensor A and sensor B, and $r_{SA}$ is the distance between sensor B and the piezoelectric actuator.

The incident signal $V_{in}$ was separated from the superposed signal as follows:

(6) $$\begin{array}{ccc} V_{in} & = & V_{B}\exp\left(-jkr_{SS}\right)-V_{A}\\ & = & S\left(\omega \right)\left[P_{in}\exp\left(jkr_{SA}\right)+P_{r}\exp\left(-jkr_{SA}\right)\right]\exp\left(-jkr_{SA}\right)-S\left(\omega \right)[P_{in}\exp\left(2jkr_{SA}\right)\\ & & \;+P_{r}\exp\left(-2jkr_{SA}\right)]\\ & = & S\left(\omega \right)P_{in}\exp\left(jkr_{SA}\right)\left[\exp\left(-jkr_{SA}\right)-\exp\left(jkr_{SA}\right)\right]\\ & = & -2jS\left(\omega \right)P_{in}\sin\left(kr_{SA}\right)\exp\left(jkr_{SA}\right). \end{array}$$

Furthermore, the reflected signal $V_{r}$ can be expressed as

(7) $$\begin{array}{ccc} V_{r} & = & V_{A}\exp\left(-jkr_{SS}\right)-V_{B}\\ & = & S\left(\omega \right)\left[P_{in}\exp\left(2jkr_{SA}\right)+P_{r}\exp\left(-2jkr_{SA}\right)\right]\exp\left(-jkr_{SA}\right)\\ & & \;-S\left(\omega \right)\left[P_{in}\exp\left(jkr_{SA}\right)+P_{r}\exp\left(-jkr_{SA}\right)\right]\\ & = & -2jS\left(\omega \right)P_{r}\sin\left(kr_{SA}\right)\exp\left(-2jkr_{SA}\right). \end{array}$$

**Figure 11:**
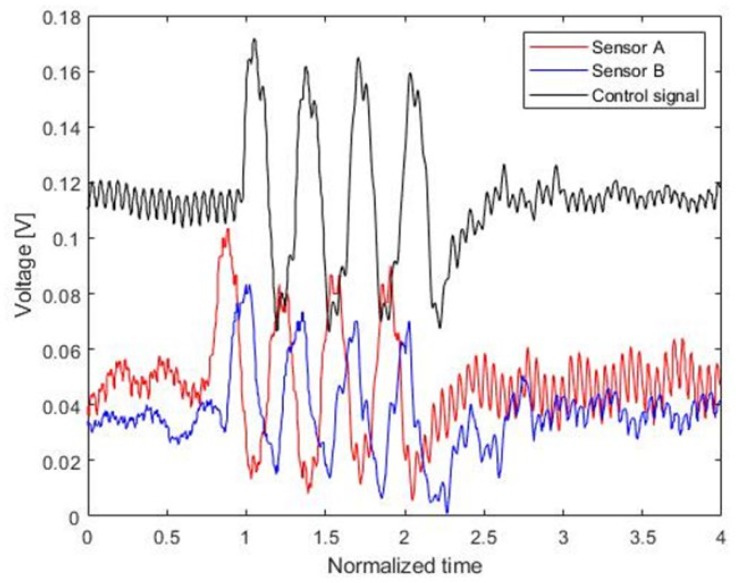
Control signal and PVDF signal.

As shown in Figure 11, each PVDF signal shows the phase shift, which depends on the sensor structure. The control signal drives the piezoelectric actuator when the reflected signal is incident on the surface of the piezoelectric actuator.

### 4.3. System Control Parameter

The reflected acoustic signal $P_{r}$ was estimated by multiplying the incident acoustic signal $P_{in}$, which propagates to the actuator surface, with the reflection coefficient R [13]:

(8) $$P_{r}=R\times P_{in}.$$

The control acoustic signal is given by

(9) $$P_{c}=V_{in}exp\left(-jkr_{SA}\right)\times G\times T\left(\omega \right),$$

and the total reflected signal can be obtained from Equations (8) and (9) as follows:

(10) $$\begin{array}{ccc} \bar{P_{r}} & = & P_{r}+P_{c}=R\times P_{in}-2jSP_{in}sin\left(kr_{SA}\right)\times G\times T\left(\omega \right),\\ & = & P_{in}\left[R+2SC_{0}\omega sin\left(\frac{\omega }{c}r_{SA}\right)\times G\right]. \end{array}$$

The system control parameter G was set to $G_{0}$, which is the value that makes the amplitude of the total reflected signal zero at a specific frequency ($\omega_{0}$). $G_{0}$ can be expressed as

(11) $$G_{0}=\frac{-R}{2SC_{0}\omega_{0}\sin\left(\frac{\omega_{0}}{c}r_{SA}\right)}.$$

$G_{0}$ is approximately linear in the low-frequency range considered in this study, and the range included the resonant frequency of the piezoelectric actuator. The echo reduction rate of the piezoelectric acoustic absorber system was defined as the ratio between the amplitude of the source signal and the total reflected signal on the logarithmic scale:

(12) $$Echo_{R}=20\;\log_{10}\left|\frac{\bar{P_{r}}}{P_{in}}\right|.$$

## 5. Real-Time Gain Tuning of Adaptive Controller

**Figure 12:**
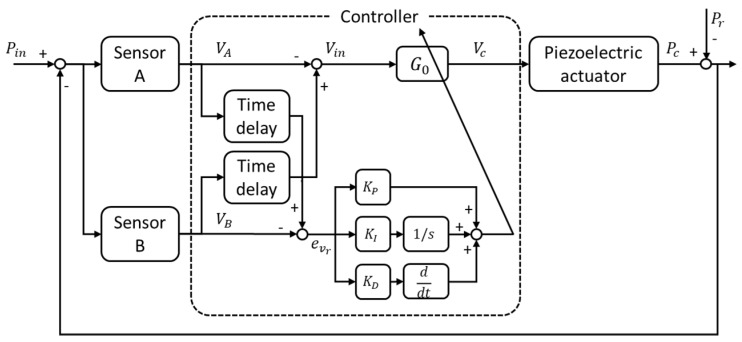
Block diagram of the proposed adaptive feedback control scheme.

Owing to the characteristics of the sensor system, it is difficult to completely eliminate the echo by setting a constant gain. A constant gain cannot appropriately respond to variations in the characteristics with frequency. Accordingly, this study used adaptive control signals to attenuate the reflected wave. The system control parameters were investigated over a range of frequencies to enhance the reduction performance, and an adaptive feedback control scheme, shown in Figure 12, was used. First, an appropriate $G_{0}$ was defined for the frequency of the incident wave in real time. Second, the real-time control parameter (α) was updated by using a proportional–integral–derivative control scheme and the error in the reflected signal, which was estimated by employing the time-delay signal separation method, was compensated for. The optimal values of $K_{P}$ (0.0393), $K_{I}$ (0.0212), and $K_{D}$ (0.0071) were obtained through wave reduction experiments.

(13) $$V_{c}=G_{0}\times V_{in}\left(1+\alpha \right),$$

(14) $$\alpha =K_{P}\times e_{V_{r\left(t\right)}}+K_{I}\times \int e_{V_{r\left(t\right)}}dt+K_{D}\times \frac{de_{V_{r\left(t\right)}}}{dt}.$$

## 6. Echo Reduction Experiments

**Figure 13:**
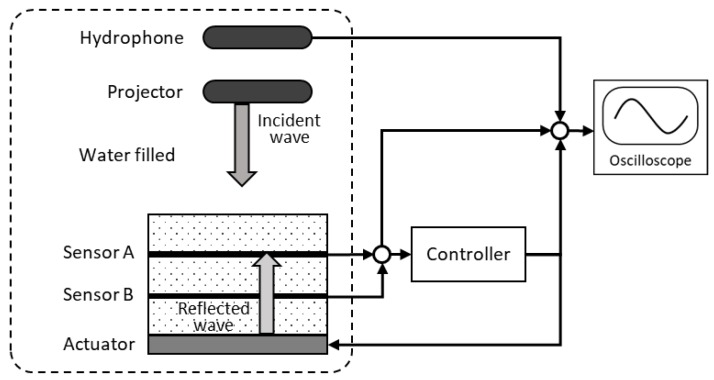
Setup of the echo reduction experiment.

An echo reduction experiment was conducted to evaluate the wave reduction of the multilayer acoustic absorber system. The experimental setup is shown in Figure 13. A projector (TC4013) generated the source signal and a hydrophone (TC4013) collected the reflected signal; these two components were placed 150 and 200 mm from the smart skin acoustic absorber system, respectively.

First, an experiment involving an active control approach was conducted. For the verification, the performance of a multilayer acoustic absorber employing the active control scheme for wave cancellation was compared with that of a multilayer acoustic absorber without any control scheme (passive control).

**Figure 14:**
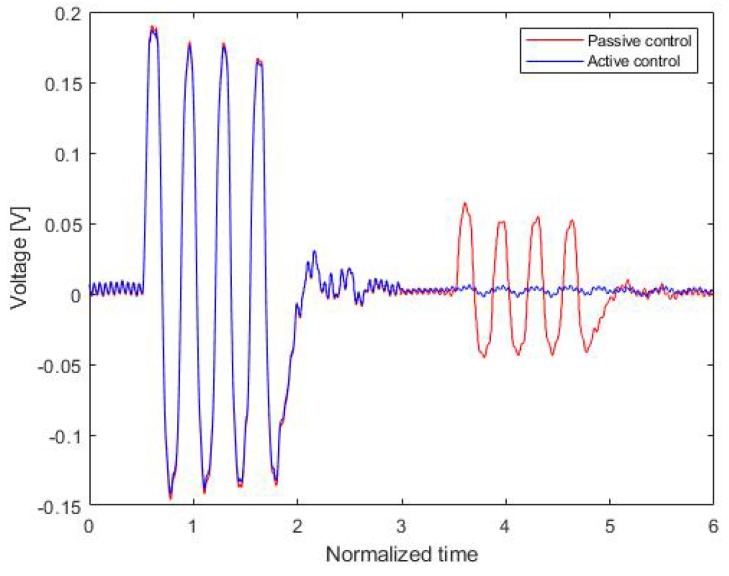
Time variation of the measured signal.

Figure 14 shows a comparison of the acoustic signal waveforms measured by the hydrophone with and without the active control scheme. The source signal was a four-cycle tone-burst sinusoidal signal. Its amplitude was 168 mV, while the amplitude of the reflected signal with passive control was 55 mV. This implied that the passive echo reduction rate of the proposed sensor system was −9.7 dB. By contrast, for active control with a constant control parameter, the reflected signal was about 5 mV and the echo reduction rate was −31.1 dB.

**Figure 15:**
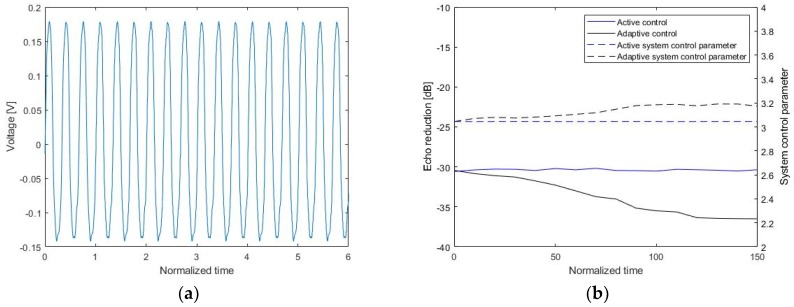
Use of active noise control: (**a**) a continuous input signal and (**b**) the echo reduction rate and system control parameter.

Figure 15 shows an input signal on project and outputs measured by the hydrophone. Figure 15a shows a continuous sinusoidal source signal at a low frequency, and Figure 15b presents a comparison of echo reduction in the proposed multilayer sensor between the active control and the adaptive control schemes. When active control was used, $G_{0}$ was fixed, and echo reduction was about −31.1 dB. In the adaptive control approach, the control signal $V_{c}$ varied with the updated real-time control parameter according to Equations (13) and (14). The final echo reduction improved from −31.1 dB to −36.5 dB over time. Thus, the use of the adaptive parameter (α) updated in real time showed better reduction performance in the multilayer piezoelectric sensor.

**Figure 16:**
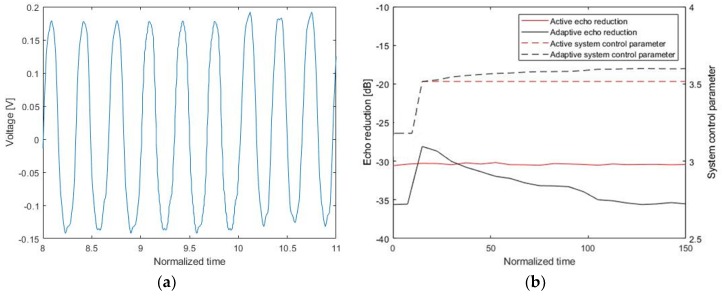
Adaptive control for a change of frequency: (**a**) a continuous input signal and (**b**) the echo reduction rate and system control parameter.

The echo reduction experiment was conducted by changing the frequency of the source signal at 10 s. Figure 16 shows an input signal on the project and the output of the hydrophone, respectively. The echo reduction is compared between the active control and the adaptive control schemes. The echo reduction achieved by the active control scheme is about −31.1 dB. With the adaptive control scheme, the echo reduction initially decreased to approximately −29 dB at 10 s and then increased to −36 dB. The system control parameter responded to a change in the input signal (at 10 s) in real time. As shown in Figure 15b, the adaptive control parameter provided higher echo reduction compared to the fixed control parameter in active control.

## 7. Conclusions

This paper presents a multilayer piezoelectric acoustic absorber system with high echo reduction at low frequencies. Despite the separation of the incident signal from the two sensor signals through a time-delay signal separation method, the absorber system cannot respond precisely to environmental changes. The use of the proposed real-time system control parameter in the multilayer acoustic absorber system enables the system to maintain a high echo reduction performance when the environment changes. Simulation results for the smart skin acoustic absorber system showed the possibility of high echo reduction at low frequencies. In an experiment conducted in a water tank, the multilayer piezoelectric acoustic absorber system with an adaptive controller could attenuate reflected waves at low frequencies.

This study examined the attenuation in the multilayer acoustic absorber system for vertically incident 1D acoustic signals. A future study will focus on echo reduction for obliquely incident signals through the use of multiple acoustic absorber systems.

## Figures and Tables

**Table 1:** Properties of the components of the proposed multilayer sensor.

	Wave Speed (m/s)	Density $\left(kg/m^{3}\right)$	Piezoelectric Coefficients $\left(10^{-12}\;C/N\right)$	Coupling Coefficients
PIN–PMN–PT composite	3190	3150	$d_{22}$ = 994	$k_{33}=0.843$
PVDF	1410	1780	$d_{33}=-30$	$k_{33}=0.2$
Rho-c rubber	1600	980	-	-
